# Overexpression of Cullin7 is associated with hepatocellular carcinoma progression and pathogenesis

**DOI:** 10.1186/s12885-017-3839-7

**Published:** 2017-12-06

**Authors:** Jun An, Zhigang Zhang, Zhiyong Liu, Ruizhi Wang, Dayang Hui, Yi Jin

**Affiliations:** 1 Department of Cardiothoracic Surgery, the Third Affiliated Hospital, Sun Yat-Sen University, Guangzhou, China; 2 Department of Pathology, Guangdong Provincial Key Laboratory of Liver Disease Research, the Third Affiliated Hospital, Sun Yat-Sen University, 600 Tianhe Road, Guangzhou, Guangdong 510630 China; 3 Department of Emergency Medicine, the Third Affiliated Hospital, Sun Yat-Sen University, Guangzhou, China; 4 Department of Clinical Laboratory, the First Affiliated Hospital, Sun Yat-Sen University, Guangzhou, China

**Keywords:** Cullin7, Hepatocellular carcinoma, Proliferation, Invasion, Western blot

## Abstract

**Background:**

Overexpression of Cullin7 is associated with some types of malignancies. However, the part of Cullin7 in hepatocellular carcinoma remains unclear. The aim of this study was to investigate the role of Cullin7 in pathogenesis and the progression of hepatocellular carcinoma.

**Methods:**

In the present study, the expression of Cullin7 in hepatocellular carcinoma cell lines and five surgical hepatocellular carcinoma specimens was detected with quantitative reverse transcription PCR and western blotting. In addition, the protein expression of Cullin7 was examined in 162 cases of archived hepatocellular carcinoma using immunohistochemistry.

**Results:**

We found elevated expression of both mRNA and protein levels of Cullin7 in hepatocellular carcinoma cell lines, and Cullin7 protein was significantly upregulated in hepatocellular carcinoma compared with paired normal hepatic tissues. The immunohistochemistry analysis revealed that overexpression of Cullin7 occurred in 69.1% of hepatocellular carcinoma samples, which was a significantly higher rate than that in adjacent normal hepatic tissue (*P* < 0.01). Statistical analysis found that overexpression of Cullin7 was significantly associated with lymph node metastasis, tumor thrombus of the portal vein and advanced clinical stage (*P* < 0.05). Furthermore, by overexpressing Cullin7 in hepatocellular carcinoma HepG2 cells, we revealed that Cullin7 could significantly enhance cell proliferation, growth, migration and invasion. Conversely, knocking down Cullin7 expression with short hairpin RNAi in hepatocellular carcinoma HepG2 cells inhibited cell proliferation, growth, migration and invasion.

**Conclusion:**

Our studies provide evidence that overexpression of Cullin7 plays an important role in the pathogenesis and progression of hepatocellular carcinoma and may be a valuable marker for hepatocellular carcinoma management.

## Background

Hepatocellular carcinoma (HCC) is one of the most common malignancies, and an increase in incidence has been reported worldwide [1]. It often occurs more frequently in men than in women in all over the world, with a man: women ratio ranging from 2:1 to 8:1 in different geographic regions [2]. HCC has a poor prognosis as a result of a low detection rate at the curable stages and a high rate of recurrence [1, 3, 4]. Despite improvements that have been made in diagnosis and multimodal treatment in the past decades, the prognosis of HCC patients remains gloomy, primarily due to its high recurrence and rate of metastasis [5–7]. HCC is usually diagnosed at an advanced stage when patients are not eligible for curative treatments. Although it is widely accepted that the numerous genes involved in tumorigenesis and tumor metastasis have been identified, the molecular mechanisms underlying these processes are not well understood, and thus far, no specific marker has been reported to allow for patient-tailored therapeutic strategies [8, 9]. Moreover, prediction of the prognosis of HCC is yet rely on traditional pathological parameters for instance neoplasm size, neoplasm grade, the presence of lymph node metastasis [10, 11]. Hence, it is of great clinical value to further understand the molecular pathogenesis of HCC and to identify appropriate biomarkers for early HCC diagnosis and prognosis as well as novel therapeutic targets.

The gene of Cullin7 is a family member of E3 ligases that take part in protein ubiquitination and tags a subgroup of proteins for further proteasomal degradation [12]. Lately, new evidence has showed that there is a close connection between overexpression of Cullin7 and carcinogenesis. It was reported by Guo and colleagues that Cullin7 can furtherance proliferation and invasion of breast cancer cell by regulating p53 protein expression. Accordingly, their study provided testimony that Cullin7 may not only be a new cancer gene in pathogenesis of breast cancer but also be a new potential therapeutic target for treatment of breast cancer [13]. Moreover, Men X et al. also displayed that Cullin7 knockdown can significantly inhibited the development and invasion of xenograft tumor in mice. Furthermore, it was found that the expression of Cullin7 was enhanced in lung cancer tissues [13]. Accordingly, it is certain that Cullin7 is an important factor contributing to proliferation and survival of lung cancer cells, and its overexpression may be conducive to the pathogenesis of lung cancer of humans [14]. Furthermore, high expression of Cullin7 was observed in epithelial ovarian cancer and that promotes cancer cells migration and invasion and correlates with poor prognosis in patients with epithelial ovarian cancer [14, 15].

Taken together, the emerging genetic evidence strongly suggests that Cullin7 expression is upregulated in some cancers, including lung cancer, epithelial ovarian cancer and breast cancer, suggesting the possibility of using Cullin7 as an indicator of carcinogenesis and progression [13–15]. However, the expression of Cullin7 in HCC and its role in hepatocarcinogenesis has not been fully elucidated [12]. The function of Cullin7 in HCC development and metastasis remains unknown.

In this study, we demonstrated that the expression of Cullin7 was upregulated in HCC tissues and cell lines. In human HCC cells, the cell proliferation was significantly accelerated when Cullin7 was overexpressed. By contrast, these properties were inhibited by Knockdown of Cullin7. Our study indicates that overexpression of Cullin7 may be a novel diagnostic biomarker in HCC and may provide the basis for identifying new therapeutic targets.

## Methods

### Ethics statement

All procedures performed in the studies involving human participants were in accordance with the ethical standards of the institutional and/or national research committee and with the 1964 Helsinki declaration and its later amendments or comparable ethical standards. This study was approved by the Clinical Research Ethics Committee of the Third Affiliated Hospital, Sun Yat-sen University. Written informed consent was obtained from all the participants, and the ethical guidelines under the Declaration of Helsinki were followed.

### Cell lines

The HCC cell lines HepG2, Hep-3B, HuH7, and SMMC-7721 and the normal hepatic cell line LO2 were obtained from the Institute of Cell Biology of the Chinese Academy of Science (Shanghai, China). All cells were grown in Dulbecco’s modified Eagle’s medium (DMEM, Gibco, USA) supplemented with 10% fetal bovine serum (FBS, HyClone, USA), 100 U/ml penicillin or 100 μg/ml streptomycin, and incubated at 37 °C in a humidified incubator with an atmosphere of 5% CO_2_.

### Patients and tissue specimens

Paraffin embedded tissue sections were obtained from archived liver samples of patients with HCC (*n* = 162) at the Third Affiliated Hospital, Sun Yat-sen University between January 1, 2008 and December 31, 2009. All patients had not been pre-treated with chemotherapy or radiotherapy. In total, 162 normal liver tissue specimens were obtained from the periphery of the cancer site and utilized as controls. Histopathological diagnosis of all specimens was confirmed by two trained pathologists. The patient cohort included 144 men and 18 women with a median age of 53 years (range 19–72 years); 162 patients showed 37 as well differentiated, 112 as moderately differentiated, and 13 as poorly differentiated. According to the TNM system from the International Association for the Study of Hepatic Cancer, 64 patients in stage A, 11 in stage B, 67 in stage C, and 20 in stage D. Five biopsies of HCC tissues and the matched adjacent non-cancerous hepatic tissues were frozen and stored in liquid nitrogen until further use.

### Quantitative real-time polymerase chain reaction (qRT-PCR)

Total RNA was extracted using TRIzol reagent (Invitrogen). As previously described [16], First-strand cDNA was synthesized with a Super ScriptIII First-Strand Synthesis System (Invitrogen). qRT-PCR for cullin7 mRNA was performed on Applied Biosystems 7500 Fast Real-Time PCR system (Applied Biosystems) using One Step SYBR PrimeScript Plus RT-PCR kit (Takara Bio, Inc., Shiga, Japan) according to the manufacturer’s instruction. The cycling conditions for real-time PCR were as follows: 95 °C for 1 min, followed by 40 cycles of 95 °C for 15 s and 60 °C for 1 min. The Cullin7-specific primers were as follows: sense, 5′-CCATCTCAGAGTCCCAACAC-3′, antisense, 5′-TTCAGCACCAC GGCATAG-3′. β-actin served as an endogenous control using the following primers: sense, 5′-GTCGTCGACACGGCTCC-3′, and antisense, 5′-TCGTCGCCCACATAGGAATC-3′. The expression levels of Cullin7 were corrected by reference to β-actin, and the relative amount of mRNA was calculated by the comparative ∆Ct method.

### Vectors and retroviral infection

Cullin7-specific small hairpin RNA (shRNA; the target sequence, 5′- TGAGATCCTAGCTGAACTG-3′) were designed and synthesized by GeneChem Co, Ltd. (Shanghai, China). The Cullin7 overexpression plasmid, Cullin7-pcDNA3.1(+), was synthesized by Life Technologies (Thermo Fisher Scientific, Inc.). HepG2 cells were transfected with 2 μg of Cullin7 shRNA or Cullin7-pcDNA3.1(+) vector using Lipofectamine 2000 (Invitrogen; Thermo Fisher Scientific, Inc.).

### Western blotting

Total protein from cells or tissues was lysed using RIPA buffer (ProMab Biotechnology, USA). The concentration of the total protein was quantified using a bicinchoninic acid (BCA) protein assay kit (Boster, China). Equal amounts of protein lysates (30 μg each lane) were separated by 10% sodium dodecyl sulfate (SDS)-polyacrylamide gel electrophoresis and transferred to nitrocellulose membranes. After being blocked with 4% dry milk, the membranes were incubated with primary antibodies against Cullin7 (Cell Signaling) and β-actin at 4 °C overnight. Then, the membrane was further incubated with the corresponding horseradish peroxidase (HRP)-conjugated secondary antibody for 2 h at room temperature. Protein bands were visualized with enhanced chemiluminescence (ECL) reagents (Pierce; Thermo Fisher Scientific Inc., Waltham, MA, USA). β-actin was used as an internal reference for relative quantification.

### Immunohistochemistry

Tumor specimens were fixed in formalin, dehydrated in an ethanol series, treated with xylene, and mounted in paraffin. Four-micrometer-thick tissue sections were deparaffinized, rehydrated, and incubated with 3% H_2_O_2_ in methanol. Subsequently, the antigen was retrieved with PH 8.0 EDTA high temperature-pressure repairing, and sections were incubated with 1% BSA. Slides were incubated with rabbit anti-human monoclonal Cullin7 antibody (dilution 1:150, Abcam) at 4 °C overnight. A normal nonimmune rabbit serum was used as a negative control. After incubation with a secondary antibody at room temperature for 60 min, slides were incubated with a streptavidin-peroxidase complex. The peroxidase reaction was developed with 3,3′-diaminobenzidine, and the slides were counterstained with hematoxylin. Sections were dehydrated, rendered transparent, covered with coverslips and sealed with neutral gum.

Tumor and normal tissue specimens were assessed by two independent pathologists. Cullin7 expression was localized predominantly in the nuclei. Sections were considered positive if expression was detected in more than 10% of the cells in tumor or normal tissue.

### MTT assay

Cells were seeded on 96-well plates at initial density of (0.2 × 10^4^ per well).At each time point, cells were stained with 100 μl sterile 3-(4,5-dimethyl-2-thiazolyl)-2,5-diphenyl-2H-tetrazolium bromide dye (0.5 mg/ml, Sigma, St Louis, MO) for 4 h at 37 °C, followed by removal of the culture medium and addition of 150 μl of dimethyl sulphoxide (Sigma). The absorbance was measured at 570 nm, with 655 nm as the reference wavelength. All experiments were performed in triplicates.

### Colony formation assay

Colony formation assay performed as previously described [17]. Briefly, Cells were seeded in triplicate (500cells per 60-mm culture dish in complete medium) and incubated for 3 weeks in a humidified incubator at 37 °C. After seeding for 3 weeks, colonies were stained with crystal violet (Sigma-Aldrich, St. Louis, MO, USA). The colony formation ability was assessed by counting the number of colonies from each of the triplicate samples by using a microscope.

### Cell invasion and motility assay

Cell invasion and motility assay was performed as described by Guo et al. [13]. Briefly, cell invasion was measured in Matrigel-coated Transwell inserts containing polycarbonate filters with 8-μm pores. The inserts were coated with 50 μL of 1 mg/mL Matrigel matrix according to the manufacturer’s recommendations. Cells (2 × 10^5^) in 200 μL of serum-free medium were plated in the upper chamber, whereas 600 μL of medium with 10% fetal bovine serum was added to the lower well. After 24 h of incubation, the top cells were removed, and the bottom cells were counted. The cells that migrated to the lower surface of the membrane were fixed in 4% paraformaldehyde and stained with 0.5% crystal violet. For each membrane, five random fields were counted at 10 × magnification. Motility assays were similar to the Matrigel invasion assays except that the Transwell insert was not coated with Matrigel.

### Wound healing assay

Wound healing assay was performed as described by Guo et al. [13]. Briefly, the different cells were respectively seeded in six-well plates at a density of 1 × 10^6^ per well with complete culture medium. When cell confluency reaches 90%, then a single wound was created using a sterile plastic pipette tip. After scratching, the cells were gently washed twice with PBS and the complete culture medium was replaced. Cell migration was photographed and the width of the wound areas were measured using an inverted microscope at different time points.

### Statistical analysis

Data were analyzed using SPSS version 13.0. (Chicago, IL, USA). The relationship between the expression of Cullin7 and the clinico-pathological parameters was evaluated by χ2 analyses. A value of *P* < 0.05 was considered statistically significant.

## Results

### Overexpression of Cullin7 in HCC cell lines

**Fig. 1:**
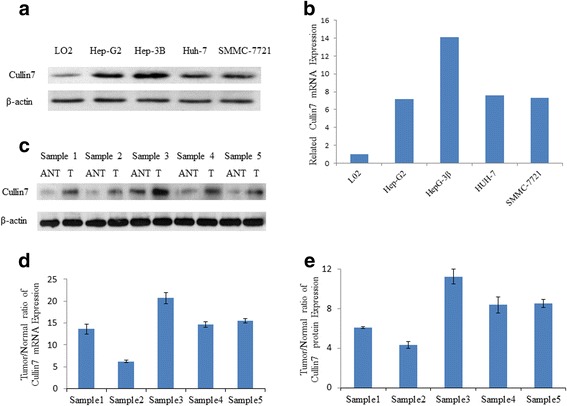
Expression of Cullin7 is elevated in HCC. **a** Expression of Cullin7 protein in the normal human hepatic cell line LO2 and in the HCC cell lines HepG2, Hep-3B, HuH71 and SMMC-7721. The expression levels were normalized to β-actin. **b** Quantification of Cullin7 mRNA in LO2 and HCC cell lines. The expression levels were normalized to β-actin. Error bars represent standard deviations calculated from three parallel experiments. **c** The expression of Cullin7 protein in each of the HCC (T) and adjacent normal hepatic tissues (ANT) determined by western blotting. **d** Real-time PCR analysis of Cullin7 expression in each of the primary HCC (T) and paired hepatic adjacent non-cancerous tissues (ANT) from the same patient. **e** Quantification of Cullin7 protein in each of the primary HCC (T) and adjacent normal hepatic tissues (ANT) determined by western blotting. The expression levels were normalized to β-actin

A western blot analysis revealed that Cullin7 protein was highly expressed in the HepG2, Hep-3B, HuH7 and SMMC-7721 HCC cell lines; however, it was weakly expressed in LO2 cells (Fig. 1a). Consistent with the upregulation of Cullin7 protein, the real-time PCR results showed that all HCC cell lines displayed significantly higher, up to 14-fold, expression of Cullin7 mRNA compared with the LO2 cell line (Fig. 1b).

### Overexpression of Cullin7 in HCC tissue

The expression of Cullin7 protein was also discovered to be increased in all five HCC tissues compared with adjacent normal hepatic tissues using western blotting (Fig. 1c). A real-time PCR analysis revealed that the tumor/normal ratio of Cullin7 mRNA expression was as high as 22-fold in one case of five paired primary HCC tissues (Fig. 1d). Furthermore, protein quantification demonstrated that four HCC tissues displayed much more than a 4-fold increase of Cullin7 protein compared with adjacent normal hepatic tissues (Fig. 1e).

### Overexpression of Cullin7 in archived HCC tissues

**Fig. 2:**
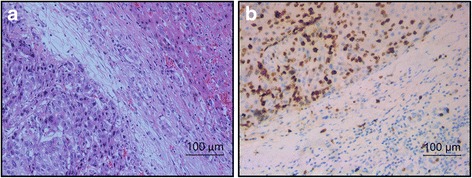
Positive expression of Cullin7 protein in HCC. **a** HE staining. The nuclei of tumor cells are large and have atypia, in contrast with those of adjacent normal hepatic tissues (HE 20 × 10). **b** Cullin7 immunohistochemical staining. Cullin7 expression is predominantly observed in the nuclei and is visualized as brown-yellow staining in HCC tissue. The expression of Cullin7 is negative in adjacent normal hepatic tissue (IHC 20 × 10)

In this study, 162 archived HCC tissues were examined using IHC staining. Immunohistochemical analysis revealed overexpression of Cullin7 predominantly in the nuclei (Fig. 2a, b). The expression of Cullin7 protein was detected in 69.1% (112/162) of HCC specimens. In contrast, Cullin7 was barely detectable and only present in 29.0% (47/162) of the normal hepatic tissues. Cullin7 was discovered to be upregulated in HCC compared with normal hepatic tissues. Statistical analyses showed that there was a striking difference in overexpression of Cullin7 between HCC and adjacent non-cancerous hepatic tissues (*P* < 0.01) (Table 1).

**Table 1:** Overexpression of Cullin7 between HCC and adjacent normal hepatic tissue

Groups	n	Cullin7	
		negative	positive (%)
HCC	162	50	112(69.1)
Normal	162	115	47 (29.0)

There was a significant difference in the expression of Cullin7 between HCC and adjacent normal hepatic tissue (*P*<0.01)

Statistical analyses were also performed to detect the correlation between Cullin7 protein expression and the clinicopathological characteristics of HCC. The results displayed that overexpression of Cullin7 protein was associated with cancer embolus, metastasis of the lymph node and the clinical stage in HCC (*P* < 0.05). However, it was not relevant to other pathological variables (*P* > 0.05) (Table 2).

**Table 2:** Relationship between overexpression of Cullin7 and clinico-pathological parameters in HCC

Parameter	n	Cullin7		
		negative(%)	positive(%)	*P*
Sex				
Male	144	44(30.6)	100(69.4)	0.810
Female	18	6(33.3)	12(66.7)	
Age(years)				
<50	97	32(33.0)	65(67.0)	0.474
≥ 50	65	18(27.7)	47(72.3)	
Tumor size(cm)				
<5	90	29(32.2)	61(67.8)	0.676
≥ 5	72	21(29.2)	51(70.8)	
Tumor numbers				
Single	116	34(29.3)	82(70.7)	0.497
multitude	46	16(34.8)	30(65.2)	
AFP(μg/L)				
<400	98	31(31.6)	67(68.4)	0.793
≥ 400	64	19(29.7)	45(70.3)	
HBsAg				
positive	135	41(30.4)	94(69.6)	0.761
negative	27	9(33.3)	18(66.7)	
Tumor thrombus				
have	69	11(15.9)	58(84.1)	0.000
no	93	39(41.9)	54(58.1)	
Lymph metastasis				
have	33	5(15.2)	28(84.8)	0.029
no	129	45(34.9)	84(65.1)	
Pathological grade				
high	37	12(32.4)	25(67.6)	0.780
moderately	112	33(29.5)	79(70.5)	
low	13	5(38.5)	8(61.5)	
Hepatic function				
Child A	106	34(32.1)	72(67.9)	0.820
Child B	36	11(30.6)	25(69.4)	
Child C	20	5(25.0)	15(75.0)	
Clinical stage				
A	64	30(46.9)	34(53.1)	0.001
B	11	5(45.5)	6(54.5)	
C	67	12(17.9)	55(82.1)	
D	20	3(15.0)	17(85.0)	

### Upregulation of Cullin7 promotes the proliferation, migration, and invasion capacities of HCC cells

**Fig. 3:**
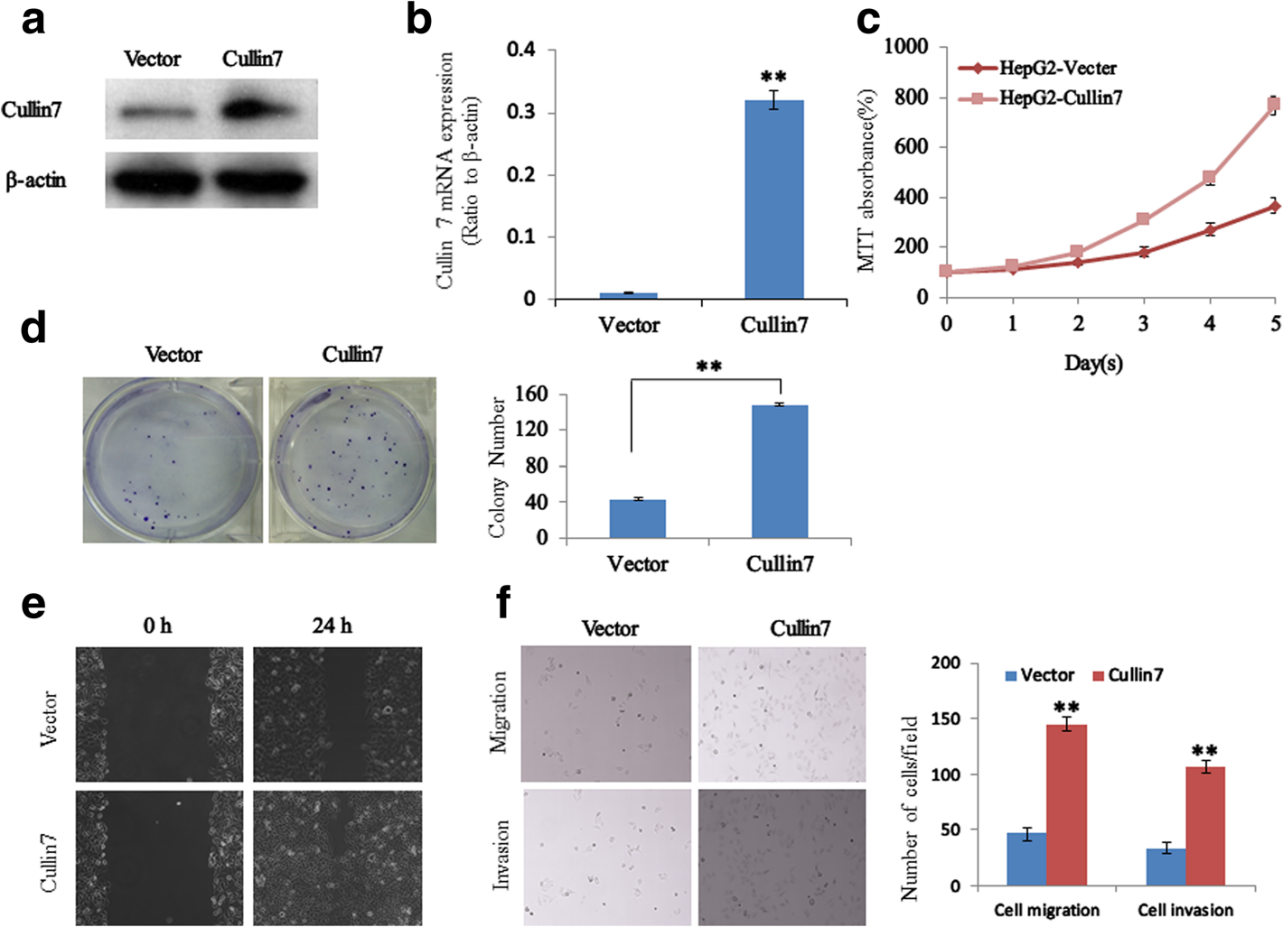
Upregulation of Cullin7 enhances the proliferation, migration, and invasion capacities of HCC cells. **a** Ectopic expression of Cullin7 in HepG2 cells analyzed by western blotting. β-actin was used as a loading control. **b** The transfection efficiency of Cullin7 was analyzed by measuring transcript levels using qRT-PCR analyses in HepG2 cells. **c** Cell proliferation after Cullin7 overexpression in cells was measured using MTT assays. **d** Colony formation assays show that upregulation of Cullin7 promotes cell growth, and the summary graphs are presented for the colony formation assay that is outlined. **e** HepG2 Cullin7 and control vector cells were subjected to a wound healing assay. **f** Overexpression of Cullin7 promoted cell invasion and migration as determined by Transwell migration and Matrigel invasion assays. Quantification of migrated cells through the membrane and invaded cells through Matrigel of each cell line are shown as a proportion of the vector controls. Error bars represent the mean ± SD of three independent experiments

To further investigate the biological role of Cullin7 expression in HCC progression, a HepG2 cell line of HCC that stably overexpressed Cullin7 was established (Fig. 3a, b). As shown in Fig. 3a and b, the mRNA and protein expression levels of Cullin7 in HepG2 cells were significantly increased compared with the vector cell lines. Furthermore, the results of MTT assays demonstrated that Cullin7-infected HepG2 cells grew faster than the control HepG2 cells by day 5 after plating (Fig. 3c). The effect of Cullin7 was also verified by the colony formation assay (Fig. 3d). Meanwhile, the influence of Cullin7 on cell migration was evaluated by a wound healing assay. The assay revealed that Cullin7-infected HepG2 cells had evidently faster closure of the wound area compared with the control HepG2 cells (Fig. 3e). HepG2 Cullin7 cells showed over two-fold more cell migration through Transwell membranes than the control cells after 24 h of incubation (Fig. 3f). HepG2 Cullin7 cells also exhibited a higher ability to invade Matrigel (Fig. 3f).

### Deregulation of Cullin7 inhibits the proliferation, migration, and invasion capacities of HCC cells

**Fig. 4:**
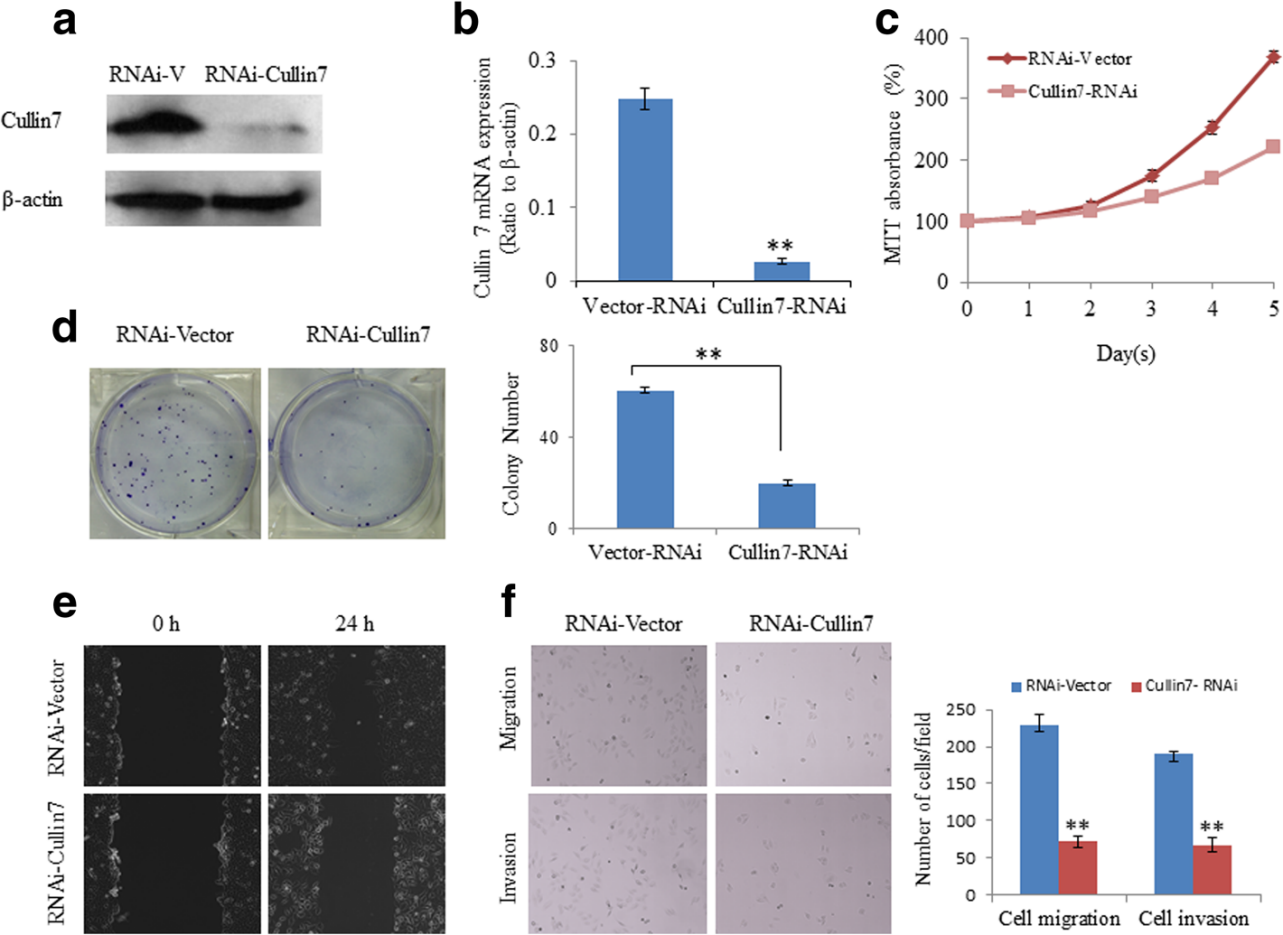
Knockdown of Cullin7 inhibited the proliferation, migration, and invasion capacities of HCC cells. **a** Knockdown of Cullin7 in specific shRNA-transduced stable HepG2 cells. β-actin was used as a loading control. **b** The transfection efficiency of shCullin7 was analyzed by measuring transcript levels using qRT-PCR analyses in HepG2 cells. **c** Silencing endogenous Cullin7 inhibited cell growth as determined using MTT assays. **d** Silencing endogenous Cullin7 inhibited cell growth as determined using colony formation assays. Summary graphs are presented for the colony formation assay that is outlined. **e** HepG2 shCullin7 and control vector cells were subjected to a wound healing assay (left panels). The uncovered areas in the wound healing assays were quantified as a percentage of the original wound area. **f** HepG2 shCullin7 and control vector cells were subjected to Transwell migration (upper panels) and Matrigel invasion assays (lower panels). Quantification of migrated cells through the membrane and invaded cells through Matrigel of each cell line are shown as a proportion of the vector controls. Error bars represent the mean ± SD of three independent experiments

The impact of Cullin7 expression on proliferation, migration and invasion was evaluated in Cullin7-knockdown cells. As shown in Fig. 4a and b, the HepG2 cells displayed significantly decreased Cullin7 expression at both the mRNA and protein levels compared with the control cells. The depletion of Cullin7 expression caused significantly compromised viability in HepG2 cells (Fig. 4c). The colony formation assay revealed that silencing endogenous Cullin7 in HepG2 cells could lead to decreases in both colony number and colony size (Fig. 4d). The effect of Cullin7 knockdown on cell migration was first assessed with a wound healing assay. Cullin7-knockdown HepG2 cells had significantly reduced closure of the wound area compared with the respective control cells (Fig. 4e). This result was confirmed using a Boyden chamber assay. HepG2 Cullin7-RNAi cells showed a lower degree of cell migration than the control cells in the Transwell migration assays (Fig. 4f). Furthermore, Cullin7-RNAi cells also exhibited a lower ability to invade Matrigel (Fig. 4f).

## Discussion

Cullin7 was initially found as a 185-kDa protein related to the large T antigen of simian virus 40 [18]. It was reported that Cullin7 not only assembles an SCF-like E3 complex that tags a subgroup of proteins for further proteasomal degradation, but also selectively interacts with the Skp.Fbw8 heterodimer [13, 19–21].

It is thought that Cullin7 promotes tumor progression through regulatory pathways involved in growth, differentiation, and apoptosis [13, 22–24]. Other studies have also shown that Cullin7 is a transcription factor and that it can induce EMT [25]. All of these findings have confirmed that overexpression of Cullin7 is associated with the occurrence and progression of cancer. However, the exact mechanisms of the involvement of Cullin7 in HCC remain unclear.

Recently, some scholars have reported the role of cullin7 in the pathogenesis and progression of HCC. Paradis et al. reported that amplification of cullin7 resulted in increased cell proliferation and demonstrated that cullin7 functions as a novel oncogene in HCC associated with metabolic syndrome [12]. Zhang D et al. also showed that Cullin7 promoted epithelial–mesenchymal transformation of cancer cells and facilitated metastasis in HCC [22].

To more deeply investigate whether upregulation of Cullin7 is also related to the progression of HCC, we performed studies to characterize the expression of Cullin7 in HCC cell lines and clinical HCC tissues.

We found that mRNA and protein expressions of Cullin7 were upregulated in HCC cell lines and HCC tissue specimens. Notably, the protein expression of Cullin7 in HCC cells and clinical HCC tissues was correlated with the mRNA expression level, indicating that the upregulation of Cullin7 in HCC may be principally created by transcriptional upregulation.

Moreover, immunohistochemical analysis demonstrated that Cullin7 was overexpressed in 69.1% of HCC tissues, while overexpression of Cullin7 was significantly lower in adjacent normal hepatic tissue (29.0%, *P* < 0.01). Furthermore, statistical analysis revealed that overexpression of Cullin7 was higher in HCC cases with lymph node metastasis, portal vein tumor thrombus and a clinical stage of C or D. Therefore, our results suggest that Cullin7 might play a role in the carcinogenesis and progression of HCC.

In order to survey the effect of overexpression of Cullin7 in proliferation, migration and invasion in HCC, the gain or loss of function of Cullin7 was detected by the help of ectopic overexpression or Cullin7 knockdown with short hairpin RNAi in HCC cell lines. Data from MTT, colony formation, wound healing, Transwell migration, and Matrigel invasion assays show that ectopic expression of Cullin7 in HepG2 cells significantly promoted cell proliferation, migration, and invasion, whereas knockdown of endogenous Cullin7 inhibited cell growth, tumor-promoting activity, migration, and invasion. The results demonstrated that Cullin7 knockdown with short hairpin RNAi can inhibit HCC cell proliferation and migration. Consequently, Cullin7 may be essential for proliferation, survival, migration and tumorigenesis of cancer cells.

Therefore, this study further confirmed the role of cullin7 in the progression and metastasis of HCC. In addition, the novelty of this study is to further clarify that cullin7 plays a role in hepatocyte migration by using wound healing assay. The protein expression of cullin7 was also detected by immunohistochemistry in 162 cases of paraffin HCC tissue samples. The results showed that cullin7 was highly expressed in HCC and the expression of cullin7 was related to lymph node metastasis, tumor thrombus and staging. To the best of our knowledge, this study is more fully and thoroughly delineated the clinical significance of the expression of Cullin7 in HCC and investigated the mechanistic role of Cullin7 in regulating HCC cell proliferation and metastasis. Taken into account together, our research results not only suggest a potential application of Cullin7 as a diagnostic indicator but also denote a relationship between the Cullin7 overexpression and the pathogenesis of HCC. As stated above, Cullin7 may play an important role in carcinogenesis, invasion and progression in HCC.

## Conclusions

In short, this study suggests that upregulation of Cullin7 may be an important factor contributing to hepatocarcinogenesis and progression, and it can be used as a diagnostic and prognostic indicator and a novel therapeutic target for HCC.

## Abbreviations

HCC: Hepatocellular carcinoma
IHC: Immunohistochemistry
qRT-PCR: Quantitative Real-time Polymerase Chain Reaction

## References

[1] Bruix J, Gores GJ, Mazzaferro V (2014). Hepatocellular carcinoma: clinical frontiers and perspectives. Gut.

[2] Li Y, Li H, Spitsbergen JM, Gong Z (2017). Males develop faster and more severe hepatocellular carcinoma than females in krasV12 transgenic zebrafish. Sci Rep.

[3] Sonohara F, Inokawa Y, Hishida M, Kanda M, Nishikawa Y, Yamada S, Fujii T, Sugimoto H, Kodera Y, Nomoto S (2016). Prognostic significance of AKR1B10 gene expression in hepatocellular carcinoma and surrounding non-tumorous liver tissue. Oncol Lett.

[4] Bu Y, Liu F, Jia QA, Yu SN (2016). Decreased expression of TMEM173 predicts poor prognosis in patients with Hepatocellular carcinoma. PLoS One.

[5] Finkelmeier F, Canli O, Tal A, Pleli T, Trojan J, Schmidt M, Kronenberger B, Zeuzem S, Piiper A, Greten FR (2016). High levels of the soluble programmed death-ligand (sPD-L1) identify hepatocellular carcinoma patients with a poor prognosis. Eur J Cancer.

[6] Dou N, Chen J, Yu S, Gao Y, Li Y (2016). G3BP1 contributes to tumor metastasis via upregulation of slug expression in hepatocellular carcinoma. Am J Cancer Res.

[7] Wang HG, Xie R, Shen P, Huang XD, Ji GZ, Yang XZ (2016). BCAT1 expression in hepatocellular carcinoma. Clin Res Hepatol Gastroenterol.

[8] Shi L, Zhang W, Zou F, Mei L, Wu G, Teng Y (2016). KLHL21, a novel gene that contributes to the progression of hepatocellular carcinoma. BMC Cancer.

[9] Zhang MH, Shen QH, Qin ZM, Wang QL, Chen X (2016). Systematic tracking of disrupted modules identifies significant genes and pathways in hepatocellular carcinoma. Oncol Lett.

[10] Han K, Kim JH, Ko GY, Gwon DI, Sung KB (2016). Treatment of hepatocellular carcinoma with portal venous tumor thrombosis: a comprehensive review. World J Gastroenterol.

[11] Grandhi MS, Kim AK, Ronnekleiv-Kelly SM, Kamel IR, Ghasebeh MA, Pawlik TM (2016). Hepatocellular carcinoma: From diagnosis to treatment. Surg Oncol.

[12] Paradis V, Albuquerque M, Mebarki M, Hernandez L, Zalinski S, Quentin S, Belghiti J, Soulier J, Bedossa P (2013). Cullin7: a new gene involved in liver carcinogenesis related to metabolic syndrome. Gut.

[13] Guo H, Wu F, Wang Y, Yan C, Su W (2014). Overexpressed ubiquitin ligase Cullin7 in breast cancer promotes cell proliferation and invasion via down-regulating p53. Biochem Biophys Res Commun.

[14] Men X, Wang L, Yu W, Ju Y (2015). Cullin7 is required for lung cancer cell proliferation and is overexpressed in lung cancer. Oncol Res.

[15] Xi J, Zeng ST, Guo L, Feng J (2016). High expression of Cullin7 correlates with unfavorable prognosis in epithelial ovarian cancer patients. Cancer Investig.

[16] Zhang B, An J, Shimada T, Liu S, Maeyama K (2012). Oral administration of Enterococcus faecalis FK-23 suppresses Th17 cell development and attenuates allergic airway responses in mice. Int J Mol Med.

[17] Sun Y, Wang Y, Fan C, Gao P, Wang X, Wei G, Wei J (2014). Estrogen promotes stemness and invasiveness of ER-positive breast cancer cells through Gli1 activation. Mol Cancer.

[18] Sarikas A, Xu X, Field LJ, Pan ZQ (2008). The cullin7 E3 ubiquitin ligase: a novel player in growth control. Cell Cycle.

[19] Ponyeam W, Hagen T (2012). Characterization of the Cullin7 E3 ubiquitin ligase--heterodimerization of cullin substrate receptors as a novel mechanism to regulate cullin E3 ligase activity. Cell Signal.

[20] Xu Y, Wang Y, Ma G, Wang Q, Wei G (2014). CUL4A is overexpressed in human pituitary adenomas and regulates pituitary tumor cell proliferation. J Neuro-Oncol.

[21] Wang Y, Ma G, Wang Q, Wen M, Xu Y, He X, Zhang P, Wang Y, Yang T, Zhan P (2013). Involvement of CUL4A in regulation of multidrug resistance to P-gp substrate drugs in breast cancer cells. Molecules.

[22] Zhang D, Yang G, Li X, Xu C, Ge H (2016). Inhibition of liver carcinoma cell invasion and metastasis by knockdown of Cullin7 in vitro and in vivo. Oncol Res.

[23] Ichikawa T, Machida N, Sasaki H, Tenmoku A, Kaneko H, Negishi R, Oi I, Fujino MA (2016). Early prediction of the outcome using tumor markers and mRECIST in Unresectable Hepatocellular carcinoma patients who underwent Transarterial Chemoembolization. Oncology.

[24] Mao J, Wang D, Wang Z, Tian W, Li X, Duan J, Wang Y, Yang H, You L, Cheng Y (2016). Combretastatin A-1 phosphate, a microtubule inhibitor, acts on both hepatocellular carcinoma cells and tumor-associated macrophages by inhibiting the Wnt/beta-catenin pathway. Cancer Lett.

[25] Fu J, Lv X, Lin H, Wu L, Wang R, Zhou Z, Zhang B, Wang YL, Tsang BK, Zhu C (2010). Ubiquitin ligase cullin 7 induces epithelial-mesenchymal transition in human choriocarcinoma cells. J Biol Chem.

